# Neuroimaging Research on Dementia in Brazil in the Last Decade: Scientometric Analysis, Challenges, and Peculiarities

**DOI:** 10.3389/fneur.2021.640525

**Published:** 2021-03-15

**Authors:** Liara Rizzi, Ítalo Karmann Aventurato, Marcio L. F. Balthazar

**Affiliations:** Department of Neurology, University of Campinas (UNICAMP), Campinas, Brazil

**Keywords:** Alzheimer's disease, Brazil, dementia, mild cognitive impaiment, MRI, neuroimaging, scientometric analysis

## Abstract

The last years have evinced a remarkable growth in neuroimaging studies around the world. All these studies have contributed to a better understanding of the cerebral outcomes of dementia, even in the earliest phases. In low- and middle-income countries, studies involving structural and functional neuroimaging are challenging due to low investments and heterogeneous populations. Outstanding the importance of diagnosing mild cognitive impairment and dementia, the purpose of this paper is to offer an overview of neuroimaging dementia research in Brazil. The review includes a brief scientometric analysis of quantitative information about the development of this field over the past 10 years. Besides, discusses some peculiarities and challenges that have limited neuroimaging dementia research in this big and heterogeneous country of Latin America. We systematically reviewed existing neuroimaging literature with Brazilian authors that presented outcomes related to a dementia syndrome, published from 2010 to 2020. Briefly, the main neuroimaging methods used were morphometrics, followed by fMRI, and DTI. The major diseases analyzed were Alzheimer's disease, mild cognitive impairment, and vascular dementia, respectively. Moreover, research activity in Brazil has been restricted almost entirely to a few centers in the Southeast region, and funding could be the main driver for publications. There was relative stability concerning the number of publications per year, the citation impact has historically been below the world average, and the author's gender inequalities are not relevant in this specific field. Neuroimaging research in Brazil is far from being developed and widespread across the country. Fortunately, increasingly collaborations with foreign partnerships contribute to the impact of Brazil's domestic research. Although the challenges, neuroimaging researches performed in the native population regarding regional peculiarities and adversities are of pivotal importance.

## Introduction

The majority of people with dementia live in low- and middle-income nations, as is the case of Brazil, the largest and the most populated country in Latin America (LA). LA is experiencing an unprecedented and fast demographic change in the last decades, with the increasing aging of the population (1). As well, Brazil has experienced significant changes in the population age pyramid. Nowadays, the country counts more than 30 million people over 60 years old (14% of the population), and by 2060 this number is projected to increase to 73 million (2). Such a consequence is the increase in the prevalence of dementia cases. In LA is expected a four-fold rise in subjects with dementia by 2050 (3). In Brazil, a recent meta-analysis, which included seven Brazilian studies, found a pooled dementia prevalence of 14.3% (6.8–23.9), but with substantial heterogeneity (4).

Neuroimaging research can provide useful diagnostic images and experimental outcomes that report and support evidence-based clinical practice (5). Moreover, is an essential part of dementia workup to exclude non-neurodegenerative causes of cognitive impairment, as well as to evaluate possible patterns of brain atrophy and cerebrovascular disease (6). Since the creation of the multicentric study Alzheimer's disease Neuroimaging Initiative (ADNI) in the United States in 2004, there was a significant increase both in the number of studies and Magnetic Resonance Imaging (MRI) techniques that have contributed to better understand the cerebral repercussions of the disease, even in the earliest phases (7). After then, different techniques have been improved, like brain volumetry (automated, manual, semi-automated), voxel-based morphometry (VBM), cortical thickness analyses, diffusion tensor imaging (DTI), and functional MRI (fMRI), especially functional connectivity, among others (8).

Outstanding the importance of neuroimaging examinations in dementia, especially in Alzheimer's disease (AD) and mild cognitive impairment (MCI), we aimed to evaluate the scientometric characteristics of Brazilian research in this field in the native population. We analyzed studies published on structural and functional neuroimaging in the last decade in a manner to assess the Brazilian scientific production in this relevant area, especially regarding original research papers. Questions addressed in this review included: journals nationalities and their impact factors, if international coauthorships, authors' gender, location of the neuroimaging research centers in Brazil, the main research funding agencies, number of publications per year, number of total citations for each paper, pathologies studied, and neuroimaging techniques utilized. Moreover, we discussed the peculiarities and challenges that this kind of research could found in a miscegenated population and a resource-limited country.

## Methods

PubMed (https://pubmed.ncbi.nlm.nih.gov/) was queried using the search strategy described in Supplementary Material 1. The results were inspected by IKA to select relevant matches. In brief, research papers were selected if they: (a) had a Brazilian author; (b) presented some kind of neuroimaging result, either quantitative or qualitative; (c) either concerned a primary or secondary neurological disease presenting with a dementia syndrome or represented cognitive aspects of the aging process; and (d) were published during or after the year of 2010 until to the date of access in the year of 2020.

Papers were classified according to their nature and design (e.g., review, longitudinal design, controlled trial), international participation in authorship, and journal nationality (Brazilian or international), first author gender, and the number of male and female authors. Web of Science (webofknowledge.com) was consulted for the number of citations received by each paper and the journal's impact factor (Journal Citation Reports™-JCR). Original research papers were further inspected and tabulated as to their MRI and other imaging methods (e.g.,18-FDG-PET), number of participants in each group (e.g., AD, MCI, controls), AD biomarker reporting, the Brazilian state where the study was performed, and funding agencies (the latter two were only accessed if the study concerned Brazilian participants).

Statistical analyses were performed using SciPy 1.5.3 (9), pandas 1.1.4 (10), and statsmodels 0.12.1 (11).

## Results

### Article Selection

**Figure 1:**
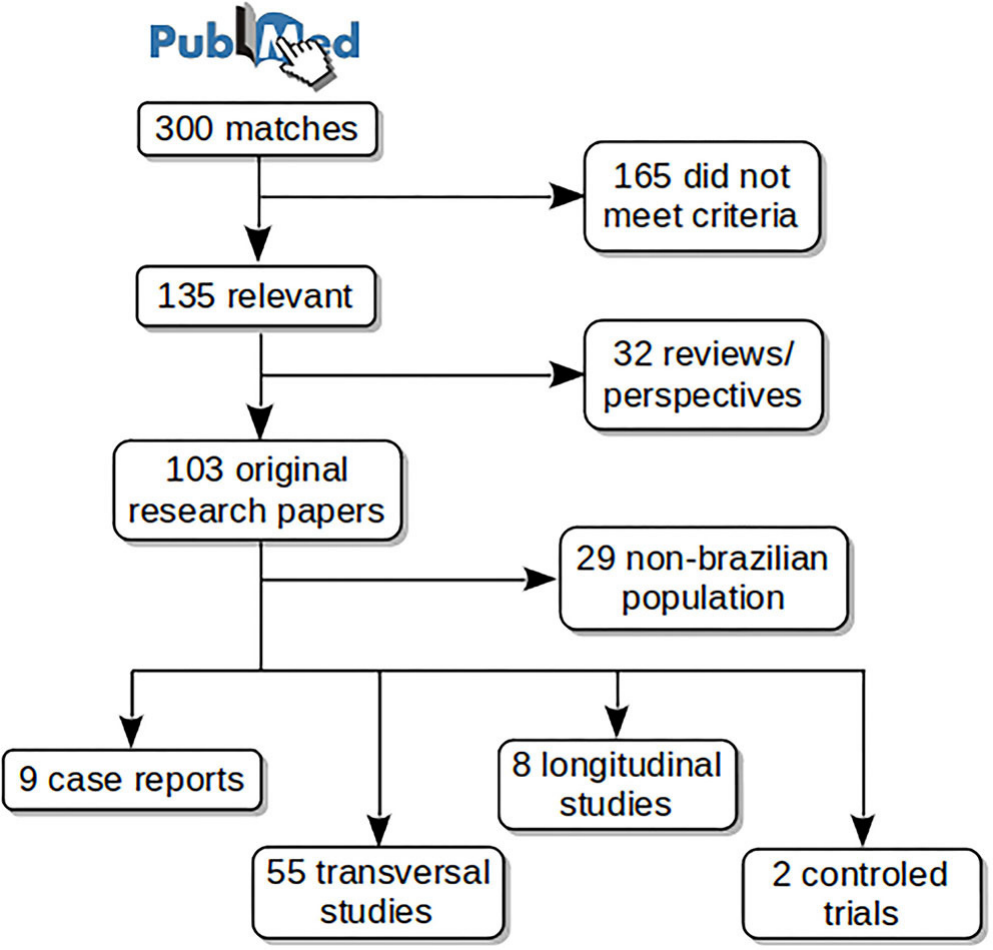
PubMed search results and article selection.

Figure 1 shows schematically the article selection process. The PubMed search resulted in 300 matches from which 135 met the aforementioned criteria. Thirty-two reviews or perspective articles were selected for a separate analysis. From the remaining 103 original research papers, 74 studied Brazilian subjects, among them: 9 case reports, 55 transversal studies, 8 longitudinal studies, and 2 controlled trials. Case reports were excluded from the main analyses. Selected articles are listed in Table 1 with the main findings, and in Supplementary Material 2 with all findings.

**Table 1:** Main findings of articles included in the present review.

**Author**	**Journal**	**Year**	**Type**	**Methods**	**Pathology**	**Reference**
Balthazar et al.	J Int Neuropsych Soc	2010	T	Morph	AD, MCI	(12)
Balthazar et al.	J Int Neuropsych Soc	2010	T	Morph	AD, MCI	(13)
Porto et al.	Dement Neuropsychol	2010	CR	Quali	PCA	(14)
Chaves et al.	J Neuroinflamm	2010	T	Morph	AD	(15)
Oliveira et al.	J Alzheimers Dis	2010	T	Morph	AD	(16)
de Toledo Ferraz Alves et al.	Curr Opin Psychiatr	2010	R			(17)
Baldaçara et al.	Rev Bras Psiquiatr	2011	L	Morph	AD, MCI	(18)
Caramelli et al.	Dement Neuropsychol	2011	P			(19)
Avila et al.	Neurobiol Aging	2011	T	Morph	Depressed Eld.	(20)
de Oliveira et al.	Am J Neuroradiol	2011	T	Morph, Other	AD, MCI	(21)
Balthazar et al.	Dement Neuropsychol	2011	T	Morph	AD, MCI	(22)
Ferreira et al.	Clinics	2011	R			(23)
Ferreira et al.	Neurobiol Aging	2011	R			(24)
de Souza et al.	Lancet	2011	CR	Quali	HAND	(25)
Caixeta et al.	Clinics	2011	CR	Quali, SPECT	PPA	(26)
Oliveira et al.	Arq Neuro-Psiquiat	2011	T	Morph, DTI	PPA	(27)
de Toledo Ferraz Alves et al.	J Alzheimers Dis	2011	T	Morph	HE	(28)
Vasconcelos et al.	Clinics	2011	T	Morph	AD	(29)
Tiel et al.	Dement Neuropsychol	2012	T	Quali	Vasc	(30)
Lanna et al.	J Neurol Sci	2012	T	Quali, SPECT	Vasc	(31)
Simon et al.	Neurosci Biobehav R	2012	R			(32)
Alves et al.	PLoS ONE	2012	T	Morph, DTI	AD, MCI	(33)
Alves et al.	Dement Neuropsychol	2012	R			(34)
Sudo et al.	Dement Neuropsychol	2012	R			(35)
Borgio et al.	Arq Neuro-Psiquiat	2012	L	Morph	MCI	(36)
Squarzoni et al.	J Alzheimers Dis	2012	T	Morph	HE	(37)
Pedro et al.	Dement Geriatr Cogn	2012	T	Morph	AD, MCI	(38)
Foss et al.	Clinics	2013	T	Morph, Other	HE	(39)
Menezes et al.	Arq Neuro-Psiquiat	2013	T	Morph, Other	AD, MCI	(40)
Radanovic et al.	Expert Rev Neurother	2013	R			(41)
Sudo et al.	Arq Neuro-Psiquiat	2013	T	Quali	MCI	(42)
Dubois et al.	Lancet Neurol	2014	P			(43)
Lee et al.	Brain	2014	T	fMRI, Morph	FTD	(44)
Teipel et al.	Psychiat Res Neuroim	2014	T	Morph	PPA	(45)
Weiler et al.	Curr Alzheimer Res	2014	T	fMRI	AD	(46)
Andrade de Oliveira et al.	J Alzheimers Dis	2014	T	Morph	AD, MCI	(47)
Weiler et al.	Brain Connectivity	2014	T	fMRI	AD, MCI	(48)
Rondina et al.	Front Aging Neurosci	2014	T	Morph	HE	(49)
Balthazar et al.	Hum Brain Mapp	2014	T	fMRI	AD	(50)
Prezzi et al.	Arq Neuro-Psiquiat	2014	CR	Quali	D-EPS	(51)
Weiler et al.	Psychiat Res Neuroim	2014	T	DTI	AD, MCI	(52)
Kilimann et al.	J Alzheimers Dis	2014	L	Morph	AD, MCI	(53)
Ferreira et al.	Rev Bras Psiquiatr	2014	R			(54)
Vasconcelos et al.	Clinics	2014	T	Morph	AD	(55)
Tovar-Moll et al.	PLoS ONE	2014	T	DTI	D-EPS, FDT	(56)
Balthazar et al.	Psychiat Res Neuroim	2014	T	fMRI	AD	(57)
de Oliveira et al.	Acta Neurol Belg	2015	CR	Quali, SPECT	FTD	(58)
Yokoyama et al.	PLOS ONE	2015	T	Morph	HE	(59)
Prado et al.	Dement Neuropsychol	2015	R			(60)
Caixeta et al.	CP & EMH	2015	T	Morph	D-EPS, FTD	(61)
Forner et al.	Neurology	2015	T	Quali	CJD	(62)
Hayata et al.	Arq Neuro-Psiquiat	2015	T	Morph	AD	(63)
da Rocha et al.	Dement Neuropsychol	2015	R			(64)
Balardin et al.	Front Aging Neurosci	2015	T	fMRI	MCI	(65)
Weiler et al.	J Alzheimers Dis	2015	L	Morph, DTI	AD	(66)
Coutinho et al.	Int Psychogeriatr	2015	T	Quali	AD, MCI	(67)
Alves et al.	BioMed Res Int	2015	R			(68)
Promteangtrong et al.	Dement Neuropsychol	2015	R			(69)
Promteangtrong et al.	Dement Neuropsychol	2015	R			(70)
Haziot et al.	Dement Neuropsychol	2015	R			(71)
Boots et al.	Arch Clin Neuropsych	2015	T	Morph	HE	(72)
Diniz et al.	Mol Psychiatr	2015	T	Morph, Ami	MCI	(73)
Agosta et al.	CNS Neurosci Ther	2015	R			(74)
Hamelin et al.	Neurob]	2015	T	Morph, Ami	AD	(75)
Grothe et al.	Cereb Cortex	2016	T	Morph, FDG	MCI	(76)
Leuzy et al.	Brain Struct Funct	2016	T	Morph, FDG, Other	FTD	(77)
Resende et al.	eNeurologicalSci	2016	T	Quali	AD, MCI	(78)
Corrêa et al.	J Mag Reson Im	2016	L	Morph, DTI	HAND	(79)
McAleese et al.	BMC Med	2016	R			(80)
Corrêa et al.	J Neuroimaging	2016	T	Morph	HAND	(81)
Teixeira et al.	AGE	2016	T	Morph, DTI	MCI	(82)
Weiler et al.	Neurosci Biobehav R	2016	R			(83)
Wang et al.	P Natl Acad Sci	2016	T	Morph	AD	(84)
Ribeiro et al.	Dement Neuropsychol	2016	R			(85)
Alves et al.	Dement Neuropsychol	2017	R			(86)
Pascoal et al.	Mol Psychiatr	2017	T	Morph, FDG, Ami	HE	(87)
Lajoie et al.	NeuroImage Clin	2017	T	fMRI, Morph	AD	(88)
Vasconcellos et al.	Parkinson's Disease	2017	T	Quali	PD	(89)
Tascone et al.	PLOS ONE	2017	T	Morph	AD	(90)
Ebadi et al.	Front Neurosci	2017	T	DTI	AD, MCI	(91)
De Souza et al.	Prion	2017	CR	Quali	CJD	(92)
Shigaeff et al.	Arch Gerontol Geriat	2017	L	fMRI	EMS	(93)
Squarzoni et al.	Clinics	2017	L	Quali	HE	(94)
Fragoso et al.	RadioGraphics	2017	R			(95)
Radanovic et al.	Dement Neuropsychol	2017	T	Quali	AD, MCI	(96)
Resende et al.	Arq Neuro-Psiquiat	2017	T	DTI	MCI	(97)
Weiler et al.	J Psychiatr Neurosci	2017	T	fMRI	AD, MCI	(98)
Rabelo et al.	Neuroradiol J	2017	T	Quali	AD, MCI	(99)
Corrêa et al.	Neuroradiol J	2017	L	fMRI, Morph, DTI	HAND	(100)
Ramos Bernardes da Silva Filho et al.	NeuroImage Clin	2017	T	Morph	AD	(101)
Swardfager et al.	Alzheimers Dement	2017	T	DTI	AD	(102)
Swardfager et al.	Neurobiol Aging	2017	T	Morph	AD	(103)
Ferreira et al.	Rev Bras Psiquiatr	2017	T	Morph, FDG, SPECT	AD	(104)
Maia da Silva et al.	Front Neurol	2017	R			(105)
Smagula et al.	Am J Geriatr Psychiat	2018	T	fMRI, Morph	HE	(106)
Branco et al.	Psychiat Res Neuroim	2018	T	Morph, DTI	MND	(107)
Simon et al.	Front Aging Neurosci	2018	CT	fMRI, Morph	MCI	(108)
Teixeira et al.	Alzheimers Dement	2018	CT	Morph	MCI	(109)
Weiler et al.	Front Aging Neurosci	2018	T	fMRI	AD, MCI	(110)
Bertrand et al.	Neuropsychology	2018	T	Morph	AD	(111)
Ventura et al.	Neuroradiol J	2018	T	fMRI	HAND	(112)
Neale et al.	NeuroImage Clin	2018	R			(113)
Miotto et al.	Neural Plast	2018	R			(114)
Axelrud et al.	Am J Psychiat	2018	T	Morph	Infants	(115)
Resende et al.	Front Aging Neurosci	2018	T	Morph	AD, MCI	(116)
Martins et al.	Dement Neuropsychol	2018	CR	Quali, SPECT	FTD	(117)
Jaswal et al.	Neurobiol Aging	2018	T	Morph	AD	(118)
Rondina et al.	NeuroImage Clin	2018	T	Morph, FDG, SPECT	AD	(119)
Magalhães et al.	Mol Neurobiol	2018	T	fMRI, Morph	AD, MCI	(120)
Swardfager et al.	Neurology	2018	T	Morph	AD, Vasc	(121)
Resende et al.	Cogn Behav Neurol	2018	T	DTI	MCI	(122)
Foss et al.	Dement Neuropsychol	2019	T	Morph	HE	(123)
Axelrud et al.	Neurobiol Aging	2019	T	fMRI	AD Relatives	(124)
Wang et al.	Commun Biol	2019	T	Morph	AD	(125)
Betts et al.	Brain	2019	R			(126)
Staffaroni et al.	Brain	2019	T	fMRI, Morph	FTD	(127)
Drummond et al.	Aging	2019	T	DTI	AD, MCI	(128)
Oliveira et al.	Dement Neuropsychol	2019	R			(129)
Schilling et al.	Mol Psychiatr	2019	T	DTI, FDG, Ami	AD, MCI	(130)
Batista et al.	Cortex	2019	T	fMRI	Vasc	(131)
Therriault et al.	Mol Neurobiol	2019	T	fMRI, Morph, Ami	AD, MCI	(132)
Ferrari et al.	Medicine	2019	L	Morph, FDG	AD	(133)
Yamashita et al.	Neuroinformatics	2019	L	Morph, FDG	AD	(134)
De Carvalho Neto et al.	Prion	2019	CR	Quali	CJD	(135)
Gonçalves et al.	Brain Res	2020	T	Morph	FTD	(136)
Martins-Filho et al.	Dement Geriatr Cogn	2020	R			(137)
Blevins et al.	Acta Neuropathol	2020	R			(138)
Rossini et al.	Clin Neurophysiol	2020	R			(139)
Dalboni da Rocha et al.	Sci Rep	2020	T	DTI	AD, MCI	(140)
Busatto Filho et al.	J Neurosci Res	2020	T	Morph, FDG, Ami	AD, MCI	(141)
Dalboni da Rocha et al.	Brain Imaging Behav	2020	T	DTI	AD, MCI	(142)
Freitas et al.	Arq Neuro-Psiquiat	2020	CR	Quali	CJD	(143)
Ducharme et al.	Brain	2020	R			(144)
Ehrenberg et al.	Alzheimers Res Ther	2020	R			(145)
Simon et al.	Int J Psychophysiol	2020	L	fMRI	MCI	(146)

*R, Review; CR, Case Report; P, Perspective; T, Transversal; L, Longitudinal; CT, Clinical Trial; AD, Alzheimer's Disease; FTD, Frontotemporal Dementia; HE, Healthy Elders; Vasc, Vascular Cognitive Impairment; MCI, Mild Cognitive Impairment; D-EPS, Dementia with extrapyramidal symptoms; CJD, Creutzfeld-Jacob Disease; EMS, Elders with metabolic syndrome; HAND, HIV Associated Neurocognitive Disorder; PPA, Primary Progressive Aphasia; PCA, Posterior Cortical Atrophy; Quali, Qualitative MRI evaluation/scales; Morph, Morphometric methods; DTI, Diffusion Tensor Imaging; fMRI, Functional MRI; FDG, [18-F]DG PET Scan; Ami, Amiloid PET Scan*.

#### Reviews

**Figure 2:**
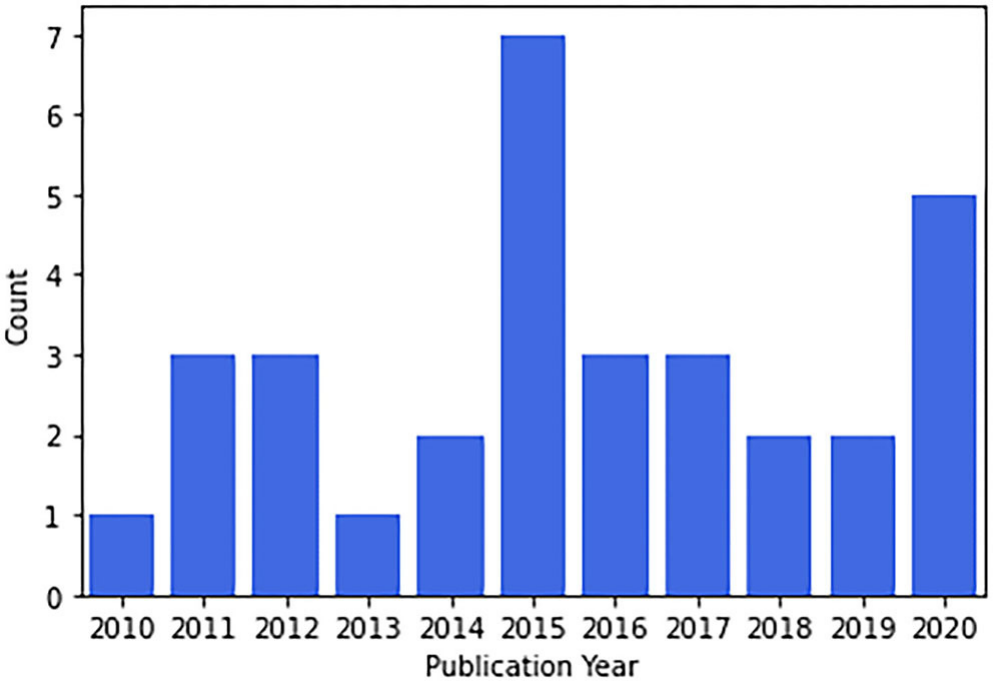
Reviews published by year.

Review papers found covered a wide range of topics. Nineteen out of 32 papers were published in non-Brazilian journals and 16/32 were coauthored by non-Brazilians. Concerning gender, males were the first authors in 20/32 papers, the median number of male and female authors were 5 and 2, respectively. Publication in international journals was correlated with international coauthorship (χ2 = 4.66, *p* = 0.031) and marginally correlated with a female first author (χ2 = 3.12, *p* = 0.077). The number of publications per year is presented in Figure 2. Time was not associated with an increasing number of publications during these years (Spearman ρ = 0.42, *p* = 0.19).

The median number of citations per article was 7 (IQR 2.75–23.75). A multivariate linear model showed a negative correlation of citation number with the Publication Year (*p* = 0.045). International Coauthorship, Journal Nationality, and First Author Gender showed no correlation. Due to the latency expected for an article to be cited, we repeated this analysis with papers published up to 2015, resulting in a median of 7 (IQR 6–33) citations. Regression results were non-significant. The journal's impact factor (JIF) was available for 21/32 papers, with a median of 4.35 (IQR 3.093–8.329). The multivariate regression showed no correlation with other variables.

#### Original Research

**Figure 3:**
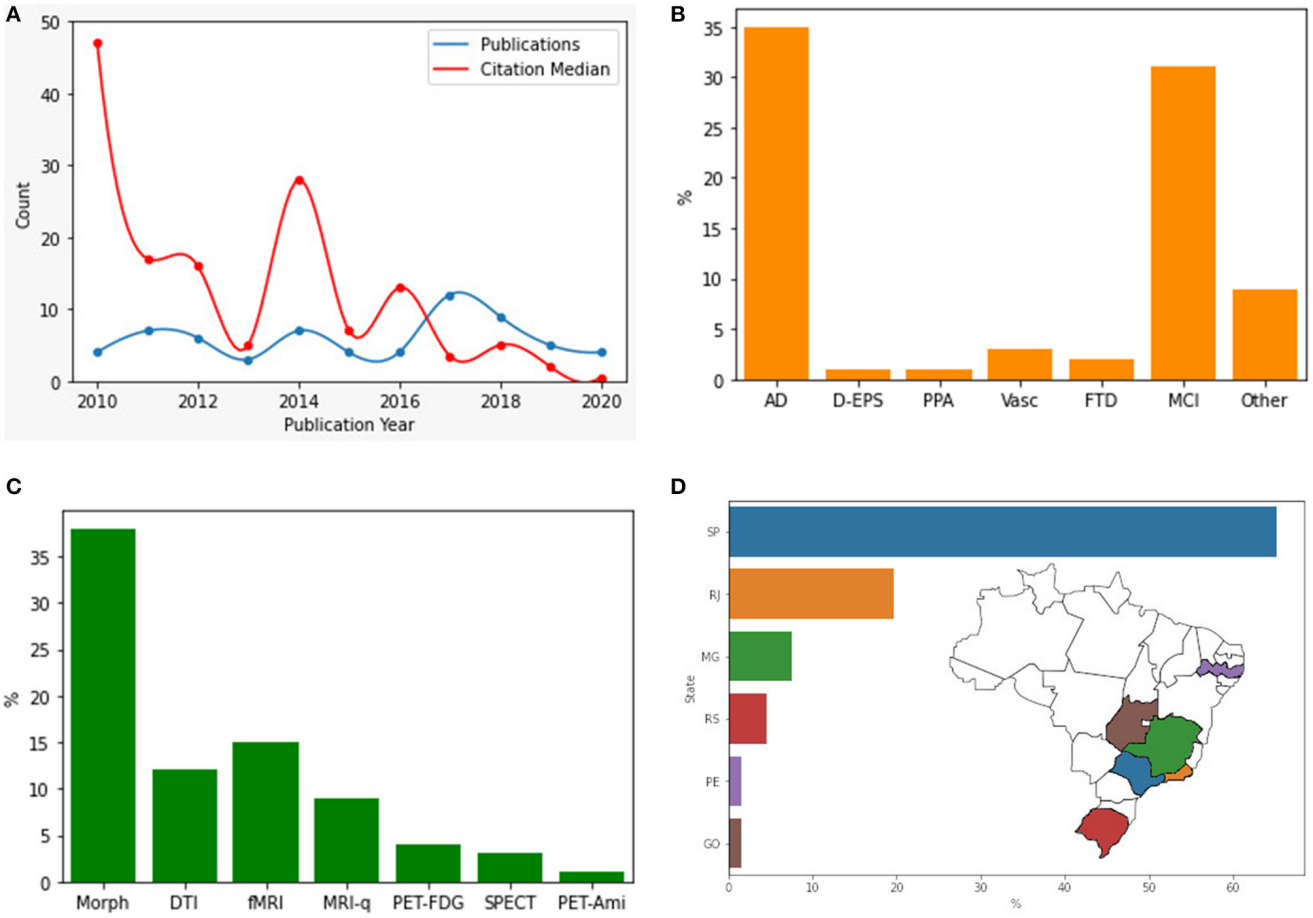
Research articles in different categories. **(A)** Original research articles published by year and citations median. **(B)** Pathology addressed by the article. **(C)** Methodology used. **(D)** Location of main neuroimaging research centers in Brazil. AD, Alzheimer's Disease; D-EPS, Dementia with extrapyramidal symptoms; PPA, Primary Progressive Aphasia; Vasc, Vascular Dementia; FTD, Frontotemporal Dementia; MCI, Mild Cognitive Impairment; Morph, Morphometric; DTI, Diffusion Tensor Imaging; fMRI, functional MRI; MRI-q, MRI qualitative analysis; PET-FDG, 18-Fluorodeoxyglucose positron emission tomography; PET-Ami: amyloid marker positron emission tomography.

Figure 3 shows the characteristics of the selected papers. Concerning the number of publications per year, there was no trend toward increasing or decreasing the number of publications (Spearman ρ = 0.13, *p* = 0.70) (Figure 3A). The most studied pathologies were AD (54%, *n* = 35) and MCI (48%, *n* = 31), followed by vascular dementia (4.6%, *n* = 3) (Figure 3B). Most studies used morphometric methods (58%, *n* = 38) followed by fMRI (23%, *n* = 15) and closely by DTI (18%, *n* = 12) (Figure 3C). Some methods addressed by only a single study nonetheless worth mentioning included spectroscopy (40), texture analysis (21), magnetization transfer ratio, and relaxometry (39).

Regarding gender analyses of original research papers, we found that females are more frequently first-authors (60%). 26/65 of the first authors are male, with a significant time effect for female authorship (Wilcoxon rank-sum test, *p* = 0.022). However, when considering all co-authors, males are more frequent (5/4 ratio). The median number of male and female authors was 5 and 4, respectively, with significantly more male authors per paper (Wilcoxon sign-rank test, *p* = 0.001). These findings might indicate that gender inequalities are less relevant in this specific field. Nineteen-out-of-sixty-five articles were co-authored by non-Brazilians. The most common nationalities among those were North-Americans (*n* = 14), British (*n* = 3), German (*n* = 2), Chilean (*n* = 2) and Swiss (*n* = 2).

There is great heterogeneity in the distribution of the research centers in the country. Research activity in Brazil has been restricted almost entirely to a few centers in the Southeast of Brazil. The vast majority of studies were set in the state of São Paulo (65%, *n* = 43), with studies also from Rio de Janeiro (20%, *n* = 13), Minas Gerais (7.6%, *n* = 5), Rio Grande do Sul (4.5%, *n* = 3), Pernambuco and Goiás (each with 1.5%, *n* = 1) (Figure 3D). Funding could be the main driver for publications. The São Paulo Research Foundation (FAPESP) was the most common funding agency, supporting 33 studies, followed by the Conselho Nacional de Desenvolvimento Científico e Tecnológico (CNPq), responsible for the funding of 28 studies, and Coordenação de Aperfeiçoamento de Pessoal do Ensino Superior (CAPES), with 14 studies being supported. Other agencies worth mentioning include Fundação de Apoio a Pesquisa do Estado do Rio de Janeiro (FAPERJ, 3 studies), Fundação de Apoio a Pesquisa do Estado de Minas Gerais (FAPEMIG, 4 studies), and the Welcome Thrust (3 studies). Seventeen studies did not report the source of resources.

The median number of citations received by original research papers was 5 (IQR 2–18). Considering only articles published up to 2015, the median was 17 (IQR 5–28). We produced three multivariate linear models to better understand what drives citation: (a) a regression for author and journal variables; (b) a regression for imaging technique; and (c) a model for the disease studied. All models were repeated restricting the sample to papers published up to 2015. The first model included Publication Year, International Coauthorship, First Author Gender, and Journal Nationality, showing a significant effect for publication in an international journal (*p* = 0.001) and the publication year (*p* < 0.001). Repeating the analysis with the papers up to 2015, only the effect of publication in an international journal remained significant (*p* = 0.037). None of the imaging techniques were associated with citation numbers either with the full or restricted sample (all *p*s non-significant). AD studies were associated with a higher number of citations (*p* = 0.003) and MCI studies showed a correlation with fewer citations (*p* = 0.04). In the restricted sample, only AD studies remained significant (*p* = 0.017).

JIF was available for 55/65 papers, with a median of 2.94 (IQR 1.90–4.35). The same models described for citations were used to predict JIF. In the first model, omitting Journal Nationality as a regressor, International Coauthorship was marginally associated with a higher JIF (*p* = 0.055). For imaging technique, Amyloid PET (*p* = 0.077) and fMRI (*p* = 0.061) showed a marginal positive correlation with JIF. None of the specific pathologies were associated with JIF.

## Peculiarities and Challenges That Hinder Neuroimaging Dementia Research in Brazil

Dementia research in low- and middle-income regions is challenging. Like other countries in LA, due to different historical processes that have occurred since the end of the fifteenth century, Brazil has its own social, cultural, racial, and regional peculiarities (147). The heterogeneity makes the diagnosis of dementia and mild cognitive impairment particularly challenging in comparison with developed countries (148). Regarding specific biological characteristics, for example, we far from understand the particularities of Brazilians miscegenated population. The regional genomic distribution of Brazilians is linked with the different colonization history of each region. Genetic admixture has been influenced by the colonization process, resulting in Brazil becoming a genetically trihybrid population (genomic inheritance of European, African, and Amerindian groups have been traced) (147). Previous epidemiological studies have highlighted that overall dementia prevalence can vary substantially across different ethnic groups and geographical regions (149). These differences in dementia prevalence rates have been attributed to different susceptibility to pathological brain changes in each ethnicity (150). In this sense, neuroimaging research in Brazil should consider these aspects. Neuroimaging studies are required to better characterize how subclinical brain changes might differ among ethnicities, and whether such differences may help explain differences in cognitive performance.

Neuroimaging research has provided evidence that previous or current adversities, such as low socioeconomic status or low levels of educational attainment, may reflect on interindividual variations in brain imaging measurements. Analysis from elderly individuals, recruited in an economically underprivileged area of São Paulo, showed reductions in both regional brain volumes and glucose metabolism in subjects with disadvantageous socioeconomic backgrounds (151, 152). Furthermore, education has a great impact on cognitive performance in older adults (153). A population census found that in 2018 nearly 52.6% of Brazilians over 25 years old did not have finished elementary school, and around 7.2% were unable to read or write (2). Variations in regional brain volumes were verified depending on the level of previous educational attainment (154). In this sense, ecological cognitive tests adapted to Brazilian characteristics (ex: including a wide range of schooling levels, illiterates, and stratified into groups of age and education) are important to be applied to more sophisticated methods, like body fluid biomarkers and neuroimaging.

Among chronic non-communicable diseases, those of the circulatory system are also the main cause of mortality worldwide, including Brazil, which has one of the highest rates in LA (155). Cerebrovascular damage, produced by midlife hypertension, diabetes, dyslipidemia, among other factors, may contribute to the onset and progression of cognitive dysfunction and dementia (156). Besides, Brazilians may have more cerebrovascular damage than other populations, as shown by Grinberg et al. (157) in a clinicopathological study with 1,291 individuals. In Brazil, cerebrovascular damage is one of the most neglected diseases, due to poor control of cardiovascular factors, especially hypertension, the main risk factor (155). In this context, it is surprising that only 4.6% of Brazilian original neuroimaging research was focused on vascular cognitive impairment. Dementia neuroimaging research in Brazil is highly focused on AD. Although AD is the most prevalent form of dementia, our results showed a disproportionate predominance to dementia epidemiology (158). The widespread interest in new drugs for AD may partially explain this finding (159). However, our study also showed that research involving AD was more likely to be cited, potentially feeding a vicious cycle. The underrepresentation of vascular dementia is particularly worrisome, as vascular risk factors and vascular pathology–either exclusive or mixed–are highly prevalent in Brazil. Once improvements in neuroimaging techniques allow detailed and sophisticated evaluation of many manifestations of cerebrovascular diseases, this topic must be considered a priority among Brazilian researchers.

The need for studies with the Brazilian population in this research field is an urgent matter. Scientific research, in general, is far from being fully developed and widespread across the country. Nowadays, even though Brazil is the 13th largest producer of research publications globally, its citation impact has historically been below the world average (160). The present work highlights some of the virtues and faults of the dementia neuroimaging research scenario in Brazil. Most of our findings are consistent with the Brazilian general scientific research background: a significant growth during the first decade of the twenty first century followed by relative stability. Furthermore, the trend toward a highly concentrated scientific production in the Southeast region along with average-to-low research impact also reflects the national tendency (160). Finally, health research is particularly affected by spatial restriction in the national territory, as the cultural, ethnic, and socioeconomic diversity is not captured by the published depictions of our reality.

Brazil has limited wherewithals, sequential financial crises, bad investment of financial resources, and a lack of priority in investing in science in the different governments. All these factors limit the quality of scientific research performed in Brazil and delay the incorporation of novelties to generate original scientific data of global relevance. One of the consequences of these facts was the failure to implement Brazilian ADNI. Lack of fundings, heterogeneity of resources, and lack of specialized centers across the different regions of the country have hampered the implementation of a large national multicenter study. Besides, only recently Brazilian researchers have started studying molecular neuroimaging, with only five amyloid PET studies, and no Tau PET studies in the last decade. Despite these difficulties, Brazilians are studying and refining new neuroimaging methods, such as functional and structural connectivity, DTI, and surface-based morphometry. Two Brazilian centers in São Paulo and Rio Grande do Sul are studying amyloid PET, and collaborative studies are taking place. Comparisons of Brazilian neuroimaging studies with other countries of Latin America are difficult, due to the lack of relevant studies in this research area as they share the same problems found in Brazil. However, our neighbor Argentina is moving forward in the field, with the establishment of the first ADNI of Latin America (161). This program currently accounts for approximately sixty participants that are evaluated by structural MRI analysis, and metabolic and amyloid PET scan (FDG and PiB). This kind of multicentric program notably will assist the development of neuroimaging studies in low- and middle-income nations in the future.

Fortunately, increasingly Brazilian researchers are working across country borders, within foreign partnerships, and the resulting papers contribute to the impact of Brazil's domestic research. Although the majority of foreign partnerships analyzed in this review were derived from North America and Europe, there are efforts to develop collaborations with our neighbors of LA. One promising group is the Latin America and Caribbean Consortium on Dementia (LAC-CD), which is a regional organization that oversees and promotes clinical and research activities on dementia. Collaborations like this certainly can set new networks to support research and increase the supply of regional and international grant proposals (162). Taken together, suggests that knowledge and technological exchange can drive the Brazilian research scenario toward a richer production. All the above-mentioned challenges require efforts toward solutions involving clinicians, researchers, and policymakers, to better understand and investigate the dementia context in a continental country such as Brazil.

## Concluding Remarks

As illustrated along with this manuscript, neuroimaging research carried out in low- and middle-income countries, such as Brazil, are challenging. Nonetheless, they are extremely important to increase the global knowledge about brain impacts derived from the inherent characteristics of the population, and their relationship with the development of dementia. Neuroimaging researches performed in the native population regarding regional peculiarities and adversities are of pivotal importance, especially in a resource-limited country facing economic and political adversities. In this sense, neuroimaging studies should address dementia not merely from a clinical perspective, but also in a societal context, considering individuals' environment and peculiarities. Despite the aforementioned limitations, Brazilian researchers in dementia should be encouraged to deepen neuroimaging studies in Alzheimer's spectrum and other prevalent conditions, such as vascular dementia.

Because our focus was neurodegenerative diseases that primarily affect cognition, we did not evaluate normal aging or other conditions that may secondarily lead to dementia, such as Parkinson's disease, Motor Neuron diseases, Epilepsy, or infectious/parasitic diseases common in Brazil. Further studies might consider the whole spectrum of dementias.

## Author Contributions

All authors contributed to the preparation and writing manuscript and approved the submitted version.

## Conflict of Interest

The authors declare that the research was conducted in the absence of any commercial or financial relationships that could be construed as a potential conflict of interest.
