# Supplementary material for: Neuroimaging Research on Dementia in Brazil in the Last Decade: Scientometric Analysis, Challenges, and Peculiarities
Source: Front Neurol. 2021 Mar 15;12:640525. doi: 10.3389/fneur.2021.640525 (PMC8005640; doi:10.3389/fneur.2021.640525)
Supplement: Supplementary file 1 [file Table_1.DOCX]

**Search Strategy**

("Dementia"[Mesh] OR "Cognitive Dysfunction"[Mesh] OR "Alzheimer Disease"[Mesh] OR "Cognitive Aging"[Mesh] OR "Frontotemporal Lobar Degeneration"[Mesh] OR "Frontotemporal Dementia" OR "Frontotemporal Lobar Degeneration" OR "Pick's Disease" OR "Lewy Body Disease"[Mesh] OR "Lewy Body Disease" OR "Lewy Body Dementia" OR "Dementia With Parkinsonism" OR "Dementia With Lewy Bodies" OR "Corticobasal Syndrome" OR "Corticobasal Degeneration" OR "Alzheimer" OR "Dementia" OR "MCI" OR "Mild Cognitive Impairment" OR "MBI" OR "Mild Behavioral Impairment" OR "HIV Associated Dementia" OR "HIV Associated Neurocognitive Disorder" OR "AIDS Dementia Complex"[Mesh] OR "Creutzfeldt-Jakob Syndrome"[Mesh] OR "Creutzfeldt-Jakob" OR "Dementia, Vascular"[Mesh] OR "Vascular Dementia" OR "Vascular Cognitive Impairment" OR "Binswanger Disease") AND ("Magnetic Resonance Imaging"[Mesh] OR "Diffusion Tensor Imaging"[Mesh] OR "Diffusion Magnetic Resonance Imaging"[Mesh] OR "MRI" OR "Magnetic Resonance Imaging" OR "fMRI" OR "Functional Connectivity" OR "DTI" OR "Diffusion Tensor Imaging" OR "VBM" OR "Volume Based Morphometry" OR "SBM" OR "Surface Based Morphometry") AND ("Brazil"[Mesh] OR "Brazil" OR "Brazilian" OR "Brasil" OR "Brasileiro" OR "Brasileira")
